# The Balance protocol: a pragmatic weight gain prevention randomized controlled trial for medically vulnerable patients within primary care

**DOI:** 10.1186/s12889-019-6926-7

**Published:** 2019-05-17

**Authors:** Miriam B. Berger, Dori M. Steinberg, Sandy Askew, John A. Gallis, Cayla C. Treadway, Joseph R. Egger, Melissa C. Kay, Bryan C. Batch, Eric A. Finkelstein, Abigail DeVries, Ashley Brewer, Gary G. Bennett

**Affiliations:** 1 Duke Global Digital Health Science Center, Duke University, Campus Box 90086, Durham, NC 27708-0086 USA; 2 Duke University School of Nursing, 307 Trent Drive, Pearson Room 2055, DUMC 3322, Durham, NC 27708 USA; 3 Duke Global Health Institute, Duke University, Trent Drive, Room 236, Durham, NC 27708 USA; 4 Duke University Medical Center, DUMC 3031, Durham, NC 27710 USA; 5 National University of Singapore, Tahir Foundation Building, 12 Science Drive 2, #10-01, Singapore, 117549 Singapore; 6 Piedmont Health Services, Inc., 127 Kingston Drive, Chapel Hill, NC 27514 USA; 7 Duke University, Department of Psychology and Neuroscience, Campus Box 90086, Durham, NC 27708 USA

**Keywords:** Obesity, Pragmatic, Digital health, E-health, Primary care, Weight, Rural health, Minority health, Latino health

## Abstract

**Background:**

For patients with obesity who are not ready for or experience barriers to weight loss, clinical practice guidelines recommend provider counseling on preventing further weight gain as a first-line treatment approach. Unfortunately, evidence-based weight gain prevention interventions are not routinely available within primary care. To address this gap, we will implement a pragmatic 12-month randomized controlled trial of a digital weight gain prevention intervention delivered to patients receiving primary care within a network of Federally Qualified Community Health Centers in central North Carolina.

**Methods:**

Balance (*Equilibrio* in Spanish) is a pragmatic effectiveness trial that will randomize adult patients who have overweight or obesity (BMI of 25–40 kg/m^2^) to either: 1) a weight gain prevention intervention with tailored behavior change goals and tracking, daily weighing on a network-connected electronic scale, and responsive weight and goal coaching delivered remotely by health center registered dietitians; or 2) a usual care program with automated healthy living text messages and print materials and routine primary care. The primary outcome will be weight gain prevention at 24-months, defined as ≤3% change in baseline weight. To align with its pragmatic design, trial outcome data will be pulled from the electronic health record of the community health center network.

**Discussion:**

For underserved, often rurally-located patients with obesity, digital approaches to promote a healthy lifestyle can curb further weight gain. Yet enrolling medically vulnerable patients into a weight gain prevention trial, many of whom are from racial/ethnic minorities, can be difficult. Despite these potential challenges, we plan to recruit a large, diverse sample from rural areas, and will implement a remotely-delivered weight gain prevention intervention to medically vulnerable patients. Upcoming trial results will demonstrate the effectiveness of this pragmatic approach to implement and evaluate a digital weight gain prevention intervention within primary care.

**Trials registration:**

NCT03003403. Registered December 28, 2016.

**Electronic supplementary material:**

The online version of this article (10.1186/s12889-019-6926-7) contains supplementary material, which is available to authorized users.

## Background

Obesity and its consequences remain at epidemic levels, particularly for medically vulnerable individuals. The associated comorbidities of obesity (i.e. diabetes, hypertension, chronic kidney disease) are particularly rampant among the medically vulnerable, who are characterized by socioeconomic disadvantage, racial/ethnic minority status, and/or residence in rural locations [1–7].

Moreover, obesity is recalcitrant to treatment [8, 9], particularly for medically vulnerable populations [10, 11]. Most trials, including primary care-based interventions, report smaller and less clinically-meaningful weight loss outcomes among such groups [10–12]. We suspect disparities in weight loss outcomes may result, in part, from limited interest in and/or motivation for weight loss within medically vulnerable groups.

In fact, up to one-half of individuals with obesity are not ready for, or interested in, weight loss [13–15], and disinterest is especially common in medically vulnerable populations [14]. In addition, motivation, a critical predictor of weight loss initiation and success [16–18], is low among participants, even in culturally-targeted interventions [19]. Both Black [20] and Latino individuals [21] have less motivation for weight change, relative to White individuals [22].

Without efficacious treatment, weight gain will likely occur. Therefore, for patients who are not ready for weight loss, clinical practice guidelines recommend that providers counsel their patients on preventing further weight gain [23]. Although weight loss reduces cardiometabolic risk [24], weight gain prevention can halt or slow progression [25–27]. This makes weight gain prevention an important part of comprehensive obesity care [3, 25–27].

However, weight gain prevention interventions are not routinely available in primary care practice. This evidence gap disproportionately affects medically vulnerable patients who have the highest rates of obesity and weight gain, but the least interest in, readiness for, and successes with weight loss. In previous work, we demonstrated successful weight gain prevention over 18 months among Black female primary care patients [28]. Here, we seek to extend those findings to an entire primary care health system, using a lower intensity intervention that has the potential to improve cost-effectiveness and dissemination potential.

The purpose of the present investigation is to conduct a 24-month pragmatic randomized controlled trial of a comprehensive, digital weight gain prevention intervention delivered in primary care practice among a medically vulnerable patient population.

## Methods/Design

In Balance (“Equilibrio” in Spanish), we will randomize 442 adults who are served at a participating community health center and classified as having overweight or obesity [Body Mass Index (BMI) of 25–40 kg/m^2^] to receive: 1) a tailored weight gain prevention intervention; or 2) a standard healthy living usual care program. The primary outcome of-interest is weight gain prevention at 24 months post-randomization, operationally-defined as ≤3% weight gain over baseline weight [29, 30], among the tailored weight gain prevention intervention arm, as compared to those receiving usual care. Secondary outcome measures include mean difference in weight change; changes in blood pressure and Framingham risk score; and intervention cost-effectiveness, based on trial results at 24-months. Approvals from the Duke Institutional Review Board and Piedmont Health Board of Directors were obtained in 2016. (See Additional file 1 for the SPIRIT figure).

### Setting

Balance will be implemented within Piedmont Health Services, Inc., a private, non-profit Federally Qualified Health Center (FQHC) network in central North Carolina. It operates 10 FQHCs that offer comprehensive family medical and dental care and integrated behavioral health, pharmacy, and support services. A meaningful-use electronic health record (EHR) system is utilized at all sites for behavioral health and medical documentation.

Piedmont Health has approximately 45 full-time equivalent primary care providers who serve more than 38,000 patients annually. The medical patient population is predominantly low-income, diverse (51% Hispanic of any race, 21% Black/African American, 3% Asian), and either uninsured or receiving public insurance (43% uninsured, 29% Medicaid and 10% Medicare). Forty-five percent of patients prefer care in a language other than English, the majority of which is Spanish, and many clinical team members are Spanish-bilingual. Piedmont Health also employs eight registered dietitians, two of whom provide medical nutrition therapy to the general health center population. Around 30% of all adult PHS medical patients have overweight (BMI of 25.0–29.9 kg/m^2^) and 47% have obesity (BMI of 30 kg/m^2^ or greater).

### Participants

Inclusion criteria for the Balance trial are as follows: currently a Piedmont Health patient at a participating health center, aged 21 years or older with a weight measured at an outpatient appointment within the last 14 days; a BMI between 25 and 40 kg/m^2^ (inclusive); and a weight of 380 pounds or less. Eligible participants must also speak English or Spanish as a primary language, have a text-enabled mobile phone and be willing to receive three to 12 weekly study-related text messages.

Due to its pragmatic design, Balance’s exclusion criteria are limited. The criteria are designed to ensure participant safety and to allow the collection of follow-up data from the Piedmont Health EHR. As such, patients will be excluded for the following reasons: being a current Piedmont Health employee; pregnancy within 12 months; lactation within two months; having given birth within the last six months; having prior or planned bariatric surgery; participating in another study to lose weight; having a cancer diagnosis and currently receiving treatment; or having a diagnosis of end stage renal disease. Participants will also be excluded if they had a cardiovascular event or hospitalization for a mental health condition within the last 12 months. Patients with a history of coronary artery revascularization within the last 12 months will be allowed to participate in the study with provider approval.

### Participant recruitment and screening

To align with the pragmatic design, participants will be recruited through in-person provider referral when seeking care at already-established appointments at Piedmont Health. Providers will speak to potentially-eligible patients about the trial during outpatient appointments, assess their interest and ask them to sign an authorization form to allow research staff to access their medical record. Research staff will then conduct a brief medical chart eligibility screen to verify if patient weight, BMI, and appointment date are within the eligible ranges. If the patient is considered eligible upon chart review, research staff will conduct a phone eligibility screening and continue with informed consent and enrollment procedures.

### Randomization

We will randomize participants to one of the two arms using permuted block randomization with stratification to balance assignment within the Piedmont Health community health centers. Randomization allocation tables will be created by a statistician using SAS software, version 9.4 (SAS Institute, Cary, NC, USA) with random block sizes ranging from 2 to 6. The tables will be stored within a secure web application at Duke University, REDCap, or Research Electronic Data Capture [31], in such a way that only the statistician will be able to view them. The random assignment of a participant will be revealed to study staff using REDCap during enrollment. Participants will be screened, enrolled, consented and randomized by research staff on the phone, and subsequently mailed study materials following their enrollment. To minimize contamination and maintain a pragmatic study design, patients who choose to enroll in the trial, but currently live in the same household as another already-enrolled Balance participant (i.e. a friend or family member), will be non-randomly assigned to the same treatment group as the initially-randomized participant. The participants assigned in this non-randomized manner will be excluded from our primary analyses but will still receive the Balance intervention. Trial outcomes will be analyzed blind to allocation status.

### Data collection

Informed by the PRECIS guidelines (PRagmatic Explanatory Continuum Indicator Summary) [32], we intentionally designed data collection procedures to maximize pragmatism. All participant baseline and follow-up data will be pulled directly from the Piedmont Health EHR and assessed at 24-months post-randomization (i.e. weights, blood pressure readings, diagnostic codes, visit notes, lab tests, medications, and appointment dates). Our intervention delivery application will be programmed to store data received from self-monitoring prompts and feedback; all inbound and outbound text messages for weight and goal coaching; and daily self-weighing measurements.

### Treatment arms

Participants will be assigned to one of two treatment arms:

### Usual care arm

Patients will receive the usual primary care offered at their health center; six months of weekly automated text messages with tips for healthy living; and print materials based on the National Heart Lung and Blood Institute’s *Aim for a Healthy Weight* [33]. Their usual primary care will not be influenced in any other way.

### Weight gain prevention intervention arm

Intervention participants will receive: 1) tailored behavior change goals through iOTA (interactive obesity treatment approach); 2) self-monitoring using connected scales and mobile technologies; 3) responsive coaching; and 4) skills training. Each of the four components is described in more detail below:

#### Tailored behavior change goals

Successful weight gain prevention requires the creation of a slight energy deficit (100–200 cal per day), representing a small change on an absolute basis. To achieve this deficit, we will utilize the interactive obesity treatment approach (iOTA). iOTA creates an energy deficit by having participants achieve simple, straightforward, and concrete behavior change goals (e.g., no fast food, no sugary drinks, walk 10,000 steps per day) [28, 34–36]. iOTA is grounded in social cognitive theory [37], from which self-efficacy will be our primary psychosocial mediator. There is strong and consistent evidence that self-efficacy is positively associated with weight-related behavior change [38–40]. Social cognitive theory indicates that behavior change can be facilitated through self-regulatory processes that we target in the intervention, including self-monitoring [41–43], goal setting [38, 44], and social support [45, 46]. iOTA has been tested in several recent primary care-based obesity treatment trials [28, 34–36, 47].

**Fig. 1:**
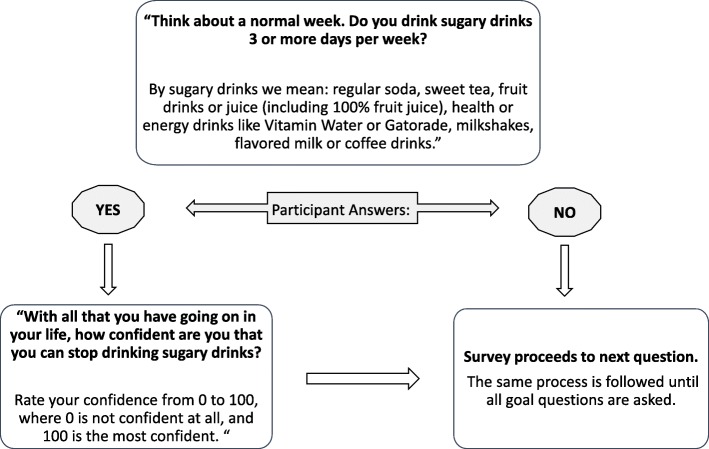
Sample iOTA survey flow for “No Sugary Drinks” behavior change goal

To assign iOTA behavior change goals, Balance participants will be administered a short survey about their current health behaviors (e.g. diet, exercise frequency, sleep, etc.). Our algorithm will rank the participants’ behaviors as high need - behaviors that promote weight gain (e.g., consumption of sugary beverages) - or low need - behaviors that create a caloric deficit (e.g., exercising 30 min at least three days per week). If participant answers are categorized as being “high need,” they will then be asked to rate their perceived confidence to change these behaviors on a scale of 0 to 100, with 0 indicating “not confident at all” and 100 indicating the “most confident” to change the behavior. (See Fig. 1 for sample survey flow.)

Based on the responses, our intervention delivery platform will use an algorithm to create a tailored list of behaviors for each participant. Each participant will then be prescribed the first four tailored behavior change goals to work on concurrently over an eight-week interval: one universal goal that all participants receive and three tailored goals. At the end of each eight-week cycle, four new goals will be assigned based on the prescribed list. At the six-month time point, we will contact participants to complete another short survey to determine the final set of eight-week goals throughout the remaining six months of the program. (See Table 1 for a complete goal list). This approach has been successfully implemented within several of our previous trials [28, 35, 47–50].

**Table 1:** Complete list of Balance behavior change goals for intervention arm

BALANCE BEHAVIOR CHANGE GOAL LIST		
	Months 1–6	Months 7–12
Universal goals assigned to all participants	Months 1–2: Weigh yourself every day Months 3–4: Walk 7000 steps a day Months 5–6: Walk 10,000 steps a day	Months 7–8: Weigh yourself every day Months 9–10: Walk 10,000 steps a day Months 11–12: Walk 10,000 steps a day
Tailored goals assigned based on iOTA survey, 3 from this list, change every 2 months	• No sugary drinks • No sweet snacks • No eating between 8 pm and 8 am^a^ • No fast food • No fried food • Eat 5+ fruits and vegetables • No salty snacks • Eat at restaurants 1 time per week or less • Watch TV < 2 h/day • Get brisk activity	• Eat 5+ fruits and vegetables • Eat red meat 1 time per week or less • Get brisk activity • Watch TV < 2 h/day • Do strength training • Eat whole grains in place of white • Eat a healthy breakfast • Sleep 7–8 h/night • No high-fat seasonings • Drink 4+ glasses water per day

^a^Computer algorithm skips assigning this goal if participant has diabetes

#### Self-monitoring and tailored feedback

##### 2a. Self-monitoring through goal tracking

**Fig. 2:**
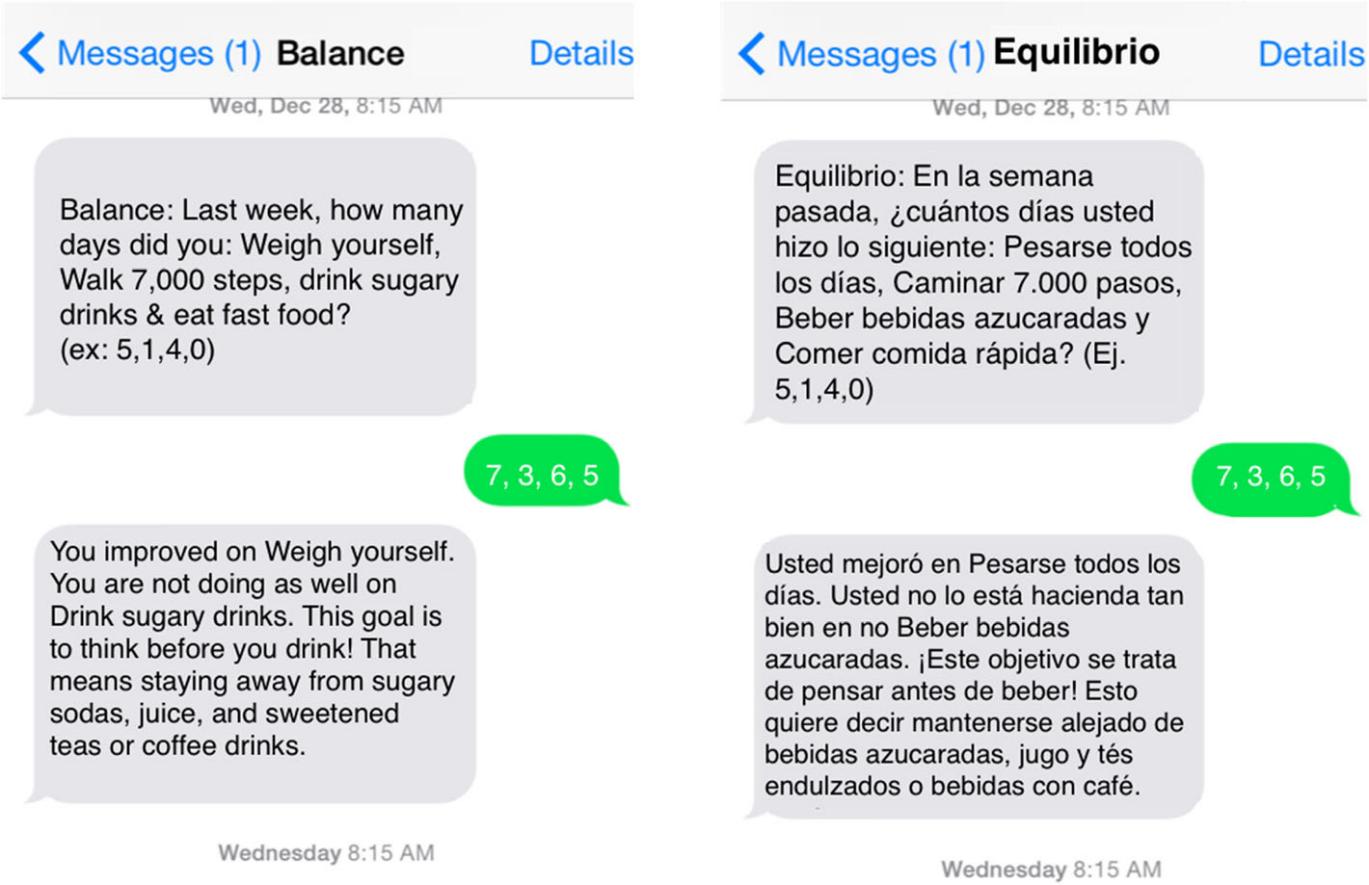
Sample Balance goal tracking text messages (English and Spanish)

Intervention participants will self-monitor adherence to their four behavior change goals each week during the 12-month intervention. To maximize engagement, we will provide opportunities for participants to self-monitor using either weekly interactive voice response (IVR) phone calls or text messages. Our IVR system will call participants and ask them to self-monitor the previous week’s goals with their keypads or voices. Utilizing a library of hundreds of hours of professionally-recorded audio files, participants will hear a human voice, rather than a digitized voice, that requests tracking data and delivers tailored feedback based on their inputted responses. (See Additional file 2 for a script of a sample IVR call). If participants do not respond to the initial call prompt, the system will send a text message 15 min later asking the goal questions. (Sample English and Spanish goal tracking text messages are depicted in Fig. 2). We have created an extensive retry protocol that continues to contact participants using both delivery channels. After our intervention delivery platform system receives a response from the participant, the system will immediately provide automated tailored feedback. Feedback messages will describe trends in progress, reinforce successes, offer motivational strategies, and short skills training tips (e.g., “Pack a healthy snack for the end of the day, when you may be tempted by fast food!”). This system has been tested previously in trials with great success in adherence to self-monitoring [28, 47–50].

##### 2b. Self-monitoring through daily self-weighing

In addition to self-monitoring behavioral goals via IVR or text, intervention participants will be asked to weigh themselves daily on a cellular-connected scale that will be shipped to participants immediately following enrollment. The scales transmit weight data directly through the cellular network; they do not require a computer, Internet, Bluetooth or wifi connection. Our intervention delivery platform will automatically receive all weight data, which will then be used to calculate average weekly weight changes for each participant. These weight data are used to trigger the responsive coaching interactions provided by Piedmont Health registered dietitians. (See Table 2). If participants do not weigh on the scale at least once weekly, they will receive weekly automated text messages reminding them to weigh (e.g., “Looks like you didn’t weigh yourself this week…”).

**Table 2:** Balance weight zones and coaching modality

Weekly Weight Zone	Average Weekly Weight Change, Compared to Baseline Weight	Coaching Mode	Response to Weight Change
GREEN	< 0 to 1.5 kg	None	Automated text messages only
YELLOW	1.6 to 2.9 kg	Coaching via text message only	Customized text messages from Balance dietitian
RED	≥ 3.0 kg	Coaching via phone call and/or text message	Balance dietitian provides counseling via phone and/or text

#### Responsive coaching

Using the weight data described above, each week, our system will automatically categorize participants into one of three intervention zones - green, yellow or red - depending on their average weight change (calculated as an average weekly weight change minus average baseline weight). The specified weight zone determines the frequency, intensity, and mode of counseling (See Table 2).

### Green zone automated  messages(<0–1.5 kg change from baseline)

Participants who are within their green zone range, i.e. maintaining their weight or having lost weight since their baseline weight, will continue to track their goals. They will receive no coaching from a Balance dietitian; rather, they will receive one automated tailored feedback text message each week congratulating them for staying within their target weight zone and providing positive reinforcement and tips to remain in the “green zone.”

### Yellow zone text counseling (+1.6–2.9 kg change from baseline)

On a daily basis, our system will automatically alert a Piedmont Health registered dietitian when a participant is in the yellow zone and needs motivational interviewing (MI)-guided counseling via text. MI enhances self-efficacy, increases recognition of inconsistencies between actual and recommended behaviors and teaches dissonance reduction skills [51]. The goal of yellow zone text counseling is to provide brief MI interactions via text to raise participant awareness about weight gain; enhance self-efficacy for behavior change; and/or to encourage problem-solving [52]. Dietitians will be trained and provided ongoing supervision and will utilize a library of special yellow zone skills training materials to counsel participants. In addition, because text messaging functionality will be built into our coaching interface, dietitians can access participant data to tailor their counseling text messages and view participant responses in real-time. Dietitians will be also be encouraged to limit their yellow zone texting frequency to a two outbound/two inbound text message exchange with participants at each coaching interval, but to use clinical discretion when more messages are needed.

### Red zone phone/text counseling (≥3.0 kg from baseline)

**Fig. 3:**
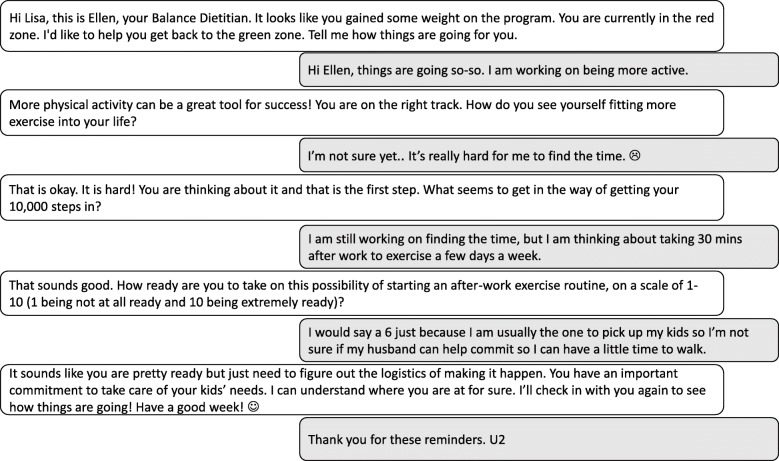
Sample red zone texting interaction between Balance registered dietitian and participant

When notified that a participant has entered the red zone, the dietitian will make a counseling call attempt and/or send a customized text message within 24 h. We will establish a maximum of 12 calls per participant over the one-year intervention for the dietitian to distribute using his/her clinical discretion. Each 15 to 20-min counseling call will be designed to assess and enhance motivation and efficacy for behavior change based on weight change and self-monitoring data, deliver in-depth behavioral skills training, and provide social support, utilizing MI principles [51, 53]. For participants who prefer texting only, a robust counseling interaction may also occur via text (See Fig. 3 for a sample red zone texting interaction).

#### Skills training

Intervention participants will receive printed skills training materials for maintaining a healthy weight, a cookbook and a pedometer to track steps. They will also receive animated videos with voiceover on how to achieve the assigned goals via DVD and YouTube links, with limited screen text for those with literacy challenges.

### Data analysis

This individually-randomized, stratified, two-arm parallel group, longitudinal trial will be powered on an unpooled two-sided chi-square test of a difference in two independent proportions, since the primary outcome is ≤3% weight gain at 24-months post randomization. We assume a difference in proportions of 0.16 (0.78 and 0.62 with ≤3% weight gain at 24 months in intervention and control participants, respectively), which is a conservative estimate based on 18-month post-baseline outcome data from our preceding work [28]. With an alpha level of 0.05, we estimate to have approximately 90% power to detect a difference in proportions of this magnitude or larger. This estimate is based on an anticipated enrollment of 442 participants, with a dropout of up to 15% of participants during the course of the study [47], resulting in a final primary outcome analysis sample size of 376 (188 per arm).

The primary analysis will be based on intention-to-treat principles where we assume that all participants have followed their randomized assignment. Clinical encounter data abstracted from Piedmont’s EHR will be the primary data source. As we have described, an outpatient visit within the last 14 days is an enrollment criterion, enabling us to capture consistently timed baseline data. However, follow-up is captured within the EHR at varying and unequal time points. As such, all statistical analyses will account for these features of the data. For all continuous outcomes, we will fit a linear mixed effects model with at least one random parameter to model the effect of time (i.e., slope) and a random intercept for person. We may also include non-linearities (e.g., splines) for time in both the random and fixed part of the models, based on data summaries, BIC, and likelihood ratio tests. Knot locations will be determined using a priori knowledge of changes in outcome over a 24-month period, as well as through graphical display of study data to estimate a smoothed function. To ensure more precise estimates through 24 months post-enrollment, we plan to abstract EHR data at least an additional 3 months (i.e., 27 months post-enrollment) on all available subjects.

The statistical model will be adjusted for Piedmont Health community health center as a fixed term in the model to account for the stratified randomization design. Given randomization, no other variables will be adjusted in the primary mixed models. Assuming a linear trajectory, the model for continuous outcomes is parameterized as:

$$ {\displaystyle \begin{array}{c}{Y}_{ij}={\upbeta}_0+{\beta}_1{trt}_i+{\beta}_2{T}_{ij}+{\beta}_3{trt}_i{T}_{ij}+{\beta}_4{CHC}_i+{b}_{0i}+{b}_{1i}{T}_{ij}+{\epsilon}_{ij},\\ {}{\epsilon}_{ij}\sim N\left(0,{\sigma}_{e_{ij}}^2\right),{b}_{0i}\sim N\left(0,{\sigma}_{b0i}^2\right),{b}_{1i}\sim N\left(0,{\sigma}_{b1i}^2\right),{\sigma}_{b_{0i},{b}_{1i}}=\mathit{\operatorname{cov}}\left({b}_{0i},{b}_{1i}\right)\end{array}} $$

where *Y*_*ij*_ is the outcome for person *i* at time *j*, *trt*_*i*_ is the intervention indicator for person *i*, *T*_*ij*_ is the continuous time (in months, with baseline as 0) for person *i* at time *j*, and *CHC*_*i*_ is the indicator of community health center for person *i*. In practice, this statistical model may be extended to allow for nonlinearities, as mentioned above.

For the primary outcome of weight gain prevention, we will compare the percentage ≤ 3% weight gain in each arm at 24 months using individual empirical best linear unbiased predictors (EBLUPs) from the mixed model. We will only use individual EBLUPs from participants with at least one EHR weight documented between 21 and 27-months post-baseline. We will compare the percentage ≤ 3% weight gain in intervention and control arms using a log-binomial model (or modified Poisson model [54] if convergence of the model is not achieved) on the EBLUP output. The exponentiated parameter estimate for intervention arm will be interpreted as the risk ratio of greater than or equal to 3% weight loss in the intervention vs. the control arm. We will also compute the risk difference estimate using a linear risk model. For all other binary outcomes, we will use a generalized linear mixed model (GLMM) with a binomial distribution and log link with similar parametrization as described above.

We will assess the intervention’s cost-effectiveness from the provider perspective. First, using an Activity Based Costing (ABC) approach [55], we will identify the key activities that drive the overall costs and assign all labor, materials and supplies, and contracted services costs to their respective activities using intervention tracking forms that we have successfully applied in prior studies [56–59]. All costs will be valued at market rates. We will also identify which costs are sunk costs (e.g., software development) and which are incremental costs as only the latter will be used in cost effectiveness analysis. This will allow for quantifying incremental per capita costs. We will then apply these costs to per capita differences in weight outcomes across arms to quantify the incremental cost per kg of weight lost (or not gained) relative to control participants. Using these estimates, we will then convert results to incremental costs per quality-adjusted life year gained (QALY) following the approach presented in Finkelstein and Verghese [60].

## Discussion

The detrimental health effects of obesity are well-documented [61–65]. As obesity and its comorbidities continue to plague our patients and health systems, there is an increasing need to provide comprehensive obesity treatment within primary care with maximal reach to patients, particularly those who reside in remote areas. Digital technologies can facilitate the reach of efficacious treatments to medically vulnerable patients [66–68], those in dire need of effective weight management programs, but with the least access to these interventions [10, 11, 69–71]. Comprehensive obesity treatment solutions that comprise both weight loss and weight gain prevention interventions are particularly needed, especially by those in medically vulnerable circumstances. Weight gain prevention is clearly recommended in obesity treatment guidelines [23], but not available or widely tested in the empirical literature. Given that up to half of the population of individuals with obesity are not attempting weight loss [13–15], weight gain prevention strategies are necessary to stem the near certainty of unhealthful weight gain, increasing the risk for morbidity and mortality.

Indeed, weight gain poses a near-inevitable threat across the lifespan among those who are not attempting weight loss. In the Coronary Artery Risk Development in Young Adults trial (CARDIA), a large national longitudinal study of 18 to 30-year-olds, only 10% of individuals could avoid weight gain over 15 years [25]. Women are at particularly high-risk for weight gain, adding as much as 10% of their initial body weight in middle age [25]. Many in medically vulnerable populations enter early adulthood having overweight or obesity and then gain weight more rapidly, and at a greater magnitude, than their lower risk counterparts [72–76]. Black women gain up to 1 kg/year, versus about 0.50–0.67 kg/year among White women [72–75, 77]. First-generation Hispanic adults gain about 0.40 kg/year, which rises to 0.85 kg/year by the second generation [78].

With weight gain a reality for so many medically vulnerable individuals, Balance is designed to be integrated within primary care, to provide a low-cost treatment option to as many adult patients as possible, in order to offer a remotely-delivered weight gain prevention strategy and healthy living content via a digital mode. To our knowledge, Balance is one of the first trials of a weight gain prevention intervention conducted within primary care. It is also one of few obesity trials to use a pragmatic approach to deliver and evaluate a digital intervention. In partnership with Piedmont Health, we will enhance our previous work to create a robust pragmatic approach to integrate a weight gain prevention treatment program into a network of community health centers that serve low-income, uninsured and underinsured patients, a large proportion who are monolingual Spanish-speakers.

The implementation of a pragmatic randomized controlled trial, such as Balance, creates an opportunity to assess the intervention’s potential generalizability to other populations and settings. In addition, we hope the trial results will demonstrate the costs and practical implications of delivering weight gain prevention treatment options for an often overburdened healthcare system. Interventions designed a priori to be integrated within practical contexts and tested using pragmatic methods may offer the best potential to improve the health and lives of those tens of millions of patients with obesity and its associated health consequences.

## Additional files

Additional file 1: SPIRIT (Standard Protocol Items: Recommendations for Interventional Trials) diagrams. This diagram shows the content of the Balance clinical trial protocol and the flow for the recruitment, screening, allocation and follow-up of all enrolled participants. (PDF 45 kb)

Additional file 2: Script of a sample IVR call. This diagram depicts a script of a sample interactive voice response call used by participants to track weekly behavior changes goals via phone. (DOCX 45 kb)

## Abbreviations

ABC: Activity based costing
BMI: Body mass index
CHC: Community health center
EBLUPs: Empirical best linear unbiased predictors
EHR: Electronic health record
FQHC: Federally-qualified health center
GLMM: Generalized linear mixed model
iOTA: Interactive obesity treatment approach
IVR: Interactive voice response
MI: Motivational interviewing
PRECIS: Pragmatic explanatory continuum indicator summary
QUALY: Quality-adjusted life year
REDCap: Research electronic data capture software

## References

[1] Ogden CL, Carroll MD, Flegal KM (2014). Prevalence of obesity in the United States. Jama..

[2] Flegal KM, Carroll MD, Kit BK, Ogden CL (2012). Prevalence of obesity and trends in the distribution of body mass index among US adults, 1999-2010. Jama.

[3] Yang L, Colditz GA (2015). Prevalence of overweight and obesity in the United States, 2007-2012. JAMA Intern Med.

[4] Ogden CL, Lamb MM, Carroll MD, Flegal KM. Obesity and socioeconomic status in adults: United States, 2005-2008. NCHS data brief. 2010;(50):1–8. 21211165

[5] Chow EA, Foster H, Gonzalez V, McIver L (2012). The disparate impact of diabetes on racial/ethnic minority populations. Clinical Diabetes.

[6] Rodriguez F, Ferdinand KC (2015). Hypertension in minority populations: new guidelines and emerging concepts. Adv Chronic Kidney Dis.

[7] Befort CA, Nazir N, Perri MG (2012). Prevalence of obesity among adults from rural and urban areas of the United States: findings from NHANES (2005-2008). J Rural Health.

[8] Svetkey LP, Stevens VJ, Brantley PJ, Appel LJ, Hollis JF, Loria CM (2008). Comparison of strategies for sustaining weight loss: the weight loss maintenance randomized controlled trial. Jama..

[9] Wadden TA, Stunkard AJ. Handbook of obesity treatment. 1st ed. New York: The Guilford Press; 2002.

[10] Osei-Assibey G, Kyrou I, Adi Y, Kumar S, Matyka K (2010). Dietary and lifestyle interventions for weight management in adults from minority ethnic/non-white groups: a systematic review. Obes Rev.

[11] Yancey AK, Kumanyika SK, Ponce NA, McCarthy WJ, Fielding JE, Leslie JP (2004). Population-based interventions engaging communities of color in healthy eating and active living: a review. Prev Chronic Dis.

[12] Wadden TA, Butryn ML, Hong PS, Tsai AG (2014). Behavioral treatment of obesity in patients encountered in primary care settings: a systematic review. Jama..

[13] Kruger J, Blanck HM, Gillespie C (2006). Dietary and physical activity behaviors among adults successful at weight loss maintenance. Int J Behav Nutr Phys Act.

[14] Wachsberg KN, Feinglass J, Williams MV, O'Leary KJ (2011). Willingness for weight loss intervention among overweight and obese inpatients. South Med J.

[15] Snook KR, Hansen AR, Duke CH, Hackney AA, Zhang J. Notice of Retraction and Replacement. Snook et al. Change in percentages of adults with overweight or obesity trying to lose weight, 1988-2014. JAMA. 2017;317(9):971-973. JAMA. 2018;320(23):2485–2486. 10.1001/jama.2018.18136. 10.1001/jama.2018.18136 30561485

[16] Webber KH, Tate DF, Michael Bowling J (2008). A randomized comparison of two motivationally enhanced internet behavioral weight loss programs. Behav Res Ther.

[17] Webber KH, Tate DF, Ward DS, Bowling JM (2010). Motivation and its relationship to adherence to self-monitoring and weight loss in a 16-week internet behavioral weight loss intervention. J Nutr Educ Behav.

[18] Williams GC, Grow VM, Freedman ZR, Ryan RM, Deci EL (1996). Motivational predictors of weight loss and weight-loss maintenance. J Pers Soc Psychol.

[19] Agurs-Collins TD, Kumanyika SK, Ten Have TR, Adams-Campbell LL (1997). A randomized controlled trial of weight reduction and exercise for diabetes management in older African-American subjects. Diabetes Care.

[20] Mattfeldt-Beman MK, Corrigan SA, Stevens VJ, Sugars CP, Dalcin AT, Givi MJ (1999). Participants' evaluation of a weight-loss program. J Am Diet Assoc.

[21] Mack KA, Anderson L, Galuska D, Zablotsky D, Holtzman D, Ahluwalia I (2004). Health and sociodemographic factors associated with body weight and weight objectives for women: 2000 behavioral risk factor surveillance system. J Women’s Health (2002).

[22] Bull FC, Eyler AA, King AC, Brownson RC (2001). Stage of readiness to exercise in ethnically diverse women: a U.S. survey. Med Sci Sports Exerc.

[23] Jensen MD, Ryan DH, Apovian CM, Ard JD, Comuzzie AG, Donato KA (2014). 2013 AHA/ACC/TOS guideline for the management of overweight and obesity in adults: a report of the American College of Cardiology/American Heart Association task force on practice guidelines and the Obesity Society. J Am Coll Cardiol.

[24] Truesdale KP, Stevens J, Cai J (2011). Differences in cardiovascular disease risk factors by weight history: the aerobics center longitudinal study. Obesity (Silver Spring, Md).

[25] Truesdale KP, Stevens J, Lewis CE, Schreiner PJ, Loria CM, Cai J (2006). Changes in risk factors for cardiovascular disease by baseline weight status in young adults who maintain or gain weight over 15 years: the CARDIA study. Int J Obes (2005)..

[26] Truesdale KP, Stevens J, Cai J (2008). Effect of 3-year weight history on blood pressure: the atherosclerosis risk in communities study. Obesity (Silver Spring, Md)..

[27] Truesdale KP, Stevens J, Cai J (2007). Nine-year changes in cardiovascular disease risk factors with weight maintenance in the atherosclerosis risk in communities cohort. Am J Epidemiol.

[28] Bennett GG, Foley P, Levine E, Whiteley J, Askew S, Steinberg DM (2013). Behavioral treatment for weight gain prevention among black women in primary care practice: a randomized clinical trial. JAMA Intern Med.

[29] Stevens J, Erber E, Truesdale KP, Wang CH, Cai J (2013). Long- and short-term weight change and incident coronary heart disease and ischemic stroke: the atherosclerosis risk in communities study. Am J Epidemiol.

[30] Stevens J, Truesdale KP, McClain JE, Cai J (2006). The definition of weight maintenance. Int J Obes (2005).

[31] Harris PA, Taylor R, Thielke R, Payne J, Gonzalez N, Conde JG (2009). Research electronic data capture (REDCap)—a metadata-driven methodology and workflow process for providing translational research informatics support. J Biomed Inform.

[32] Thorpe KE, Zwarenstein M, Oxman AD, Treweek S, Furberg CD, Altman DG (2009). A pragmatic-explanatory continuum indicator summary (PRECIS): a tool to help trial designers. J Clin Epidemiol.

[33] National Heart, Lung and Blood Institute. Aim for a healthy weight; 2005.

[34] Bennett GG, Herring SJ, Puleo E, Stein EK, Emmons KM, Gillman MW (2010). Web-based weight loss in primary care: a randomized controlled trial. Obesity (Silver Spring, Md).

[35] Bennett GG, Warner ET, Glasgow RE, Askew S, Goldman J, Ritzwoller DP (2012). Obesity treatment for socioeconomically disadvantaged patients in primary care practice. Arch Intern Med.

[36] Greaney ML, Quintiliani LM, Warner ET (2009). Weight management among patients at community health centers: the be fit be well study. Obes Manag.

[37] Bandura A (1977). Self-efficacy: toward a unifying theory of behavioral change. Psychol Rev.

[38] Elfhag K, Rossner S (2005). Who succeeds in maintaining weight loss? A conceptual review of factors associated with weight loss maintenance and weight regain. Obes Rev.

[39] Linde JA, Rothman AJ, Baldwin AS, Jeffery RW (2006). The impact of self-efficacy on behavior change and weight change among overweight participants in a weight loss trial. Int J Obes Relat Metab Disord.

[40] Richman RM, Loughnan GT, Droulers AM, Steinbeck KS, Caterson ID. Self-efficacy in relation to eating behaviour among obese and non-obese women. Int J Obes Relat Metab Disord. 2001;25(6):907–13. 10.1038/sj.ijo.0801606 11439307

[41] Boutelle KN, Kirschenbaum DS, Baker RC, Mitchell ME (1999). How can obese weight controllers minimize weight gain during the high risk holiday season? By self-monitoring very consistently. Health Psychol.

[42] Klem ML, Wing RR, McGuire MT, Seagle HM, Hill JO (1997). A descriptive study of individuals successful at long-term maintenance of substantial weight loss. Am J Clin Nutr.

[43] Levitsky DA, Garay J, Nausbaum M, Neighbors L, Dellavalle DM (2006). Monitoring weight daily blocks the freshman weight gain: a model for combating the epidemic of obesity. Int J Obes.

[44] Strecher V (2007). Internet methods for delivering behavioral and health-related interventions (eHealth). Annu Rev Clin Psychol.

[45] Stolley MR, Fitzgibbon ML, Schiffer L, Sharp LK, Singh V, Van Horn L (2009). Obesity reduction black intervention trial (ORBIT): six-month results. Obesity (Silver Spring, Md).

[46] Wing RR, Jeffery RW (1999). Benefits of recruiting participants with friends and increasing social support for weight loss and maintenance. J Consult Clin Psychol.

[47] Bennett GG, Steinberg D, Askew S, Levine E, Foley P, Batch B, et al. Effectiveness of an app and provider counseling for obesity treatment in primary care. Am J Prev Med. 2018;55(6):777–86. 10.1016/j.amepre.2018.07.005 PMC6388618 30361140

[48] Foley P, Levine E, Askew S, Puleo E, Whiteley J, Batch B (2012). Weight gain prevention among black women in the rural community health center setting: the shape program. BMC Public Health.

[49] Steinberg DM, Levine EL, Lane I, Askew S, Foley PB, Puleo E (2014). Adherence to self-monitoring via interactive voice response technology in an eHealth intervention targeting weight gain prevention among black women: randomized controlled trial. J Med Internet Res.

[50] Foley P, Steinberg D, Levine E, Askew S, Batch BC, Puleo EM (2016). Track: a randomized controlled trial of a digital health obesity treatment intervention for medically vulnerable primary care patients. Contemporary Clinical Trials.

[51] Rollnick S, Heather N, Bell A (1992). Negotiating behaviour change in medical settings: the development of brief motivational interviewing. J Ment Health.

[52] Shingleton RM, Palfai TP (2016). Technology-delivered adaptations of motivational interviewing for health-related behaviors: a systematic review of the current research. Patient Educ Couns.

[53] Webb MS, Hendricks PS, Brandon TH (2007). Expectancy priming of smoking cessation messages enhances the placebo effect of tailored interventions. Health Psychol.

[54] Zou G (2004). A modified Poisson regression approach to prospective studies with binary data. Am J Epidemiol.

[55] Baker JJ (1998). Activity-based costing and activity-based Management for Health Care.

[56] Ritzwoller DP, Glasgow RE, Sukhanova AY, Bennett GG, Warner ET, Greaney ML (2013). Economic analyses of the be fit be well program: a weight loss program for community health centers. J Gen Intern Med.

[57] Finkelstein EA, Kruger E, Karnawat S (2015). Cost-effectiveness analysis of Qsymia for weight loss. Pharmacoeconomics.

[58] Jakicic JM, Tate DF, Lang W, Davis KK, Polzien K, Rickman AD (2012). Effect of a stepped-care intervention approach on weight loss in adults: a randomized clinical trial. Jama..

[59] Finkelstein EA, DiBonaventura M, Burgess SM, Hale BC (2010). The costs of obesity in the workplace. J Occup Environ Med.

[60] Finkelstein EA, Verghese NR. Incremental cost-effectiveness of evidence-based non-surgical weight loss strategies. Clinical Obes. 2019;Apr(2):e12294. 10.1111/cob.12294 30677252

[61] Wing RR, Lang W, Wadden TA, Safford M, Knowler WC, Bertoni AG (2011). Benefits of modest weight loss in improving cardiovascular risk factors in overweight and obese individuals with type 2 diabetes. Diabetes Care.

[62] Sturm R (2002). The effects of obesity, smoking, and drinking on medical problems and costs. Health affairs (Project Hope).

[63] Bhaskaran K, Dos-Santos-Silva I, Leon DA, Douglas IJ, Smeeth L (2018). Association of BMI with overall and cause-specific mortality: a population-based cohort study of 3.6 million adults in the UK. Lancet Diabetes & Endocrinology.

[64] National Institute of Heart Lung and Blood Institute (1998). Clinical guidelines on the identification, evaluation, and treatment of overweight and obesity in adults: the evidence report.

[65] Afshin A, Forouzanfar MH, Reitsma MB, Sur P, Estep K, Lee A (2017). Health effects of overweight and obesity in 195 countries over 25 years. N Engl J Med.

[66] Huh J, Koola J, Contreras A, Castillo AK, Ruiz M, Tedone KG (2018). Consumer health informatics adoption among underserved populations: thinking beyond the digital divide. Yearbook Med Informatics.

[67] Anderson-Lewis C, Darville G, Mercado RE, Howell S, Di Maggio S (2018). mHealth technology use and implications in historically underserved and minority populations in the United States: systematic literature review. JMIR Mhealth Uhealth.

[68] Mitchell SJ, Godoy L, Shabazz K, Horn IB (2014). Internet and mobile technology use among urban African American parents: survey study of a clinical population. J Med Internet Res.

[69] Perri MG, Limacher MC, Durning PE, Janicke DM, Lutes LD, Bobroff LB (2008). Extended-care programs for weight management in rural communities: the treatment of obesity in underserved rural settings (TOURS) randomized trial. Arch Intern Med.

[70] Mejia de Grubb MC, Levine RS, Zoorob RJ (2017). Diet and obesity issues in the underserved. Primary care.

[71] Moredich CA, Kessler TA (2014). Physical activity and nutritional weight loss interventions in obese, low-income women: an integrative review. J Midwifery & Women’s Health.

[72] Boggs DA, Palmer JR, Spiegelman D, Stampfer MJ, Adams-Campbell LL, Rosenberg L (2011). Dietary patterns and 14-y weight gain in African American women. Am J Clin Nutr.

[73] Martikainen PT, Marmot MG (1999). Socioeconomic differences in weight gain and determinants and consequences of coronary risk factors. Am J Clin Nutr.

[74] Purslow LR, Sandhu MS, Forouhi N, Young EH, Luben RN, Welch AA (2008). Energy intake at breakfast and weight change: prospective study of 6,764 middle-aged men and women. Am J Epidemiol.

[75] Purslow LR, Young EH, Wareham NJ, Forouhi N, Brunner EJ, Luben RN (2008). Socioeconomic position and risk of short-term weight gain: prospective study of 14,619 middle-aged men and women. BMC Public Health.

[76] Zamora D, Gordon-Larsen P, Jacobs DR, Popkin BM (2010). Diet quality and weight gain among black and white young adults: the coronary artery risk development in Young adults (CARDIA) study (1985-2005). Am J Clin Nutr.

[77] Fowler-Brown AG, Bennett GG, Goodman MS, Wee CC, Corbie-Smith GM, James SA (2009). Psychosocial stress and 13-year BMI change among blacks: the Pitt County study. Obesity (Silver Spring, Md).

[78] Ullmann SH, Goldman N, Pebley AR (2013). Contextual factors and weight change over time: a comparison between U.S. Hispanics and other population sub-groups. Social Sci & Med (1982).

