# Supplementary material for: The Balance protocol: a pragmatic weight gain prevention randomized controlled trial for medically vulnerable patients within primary care
Source: BMC Public Health. 2019 May 17;19:596. doi: 10.1186/s12889-019-6926-7 (PMC6525404; doi:10.1186/s12889-019-6926-7)
Supplement: Supplementary file 1 — SPIRIT (Standard Protocol Items: Recommendations for Interventional Trials) diagrams. This diagram shows the content of the Balance clinical trial protocol and the flow for the recruitment, screening, allocation and follow-up of all enrolled participants. (PDF 45 kb) [file 12889_2019_6926_MOESM1_ESM.pdf]

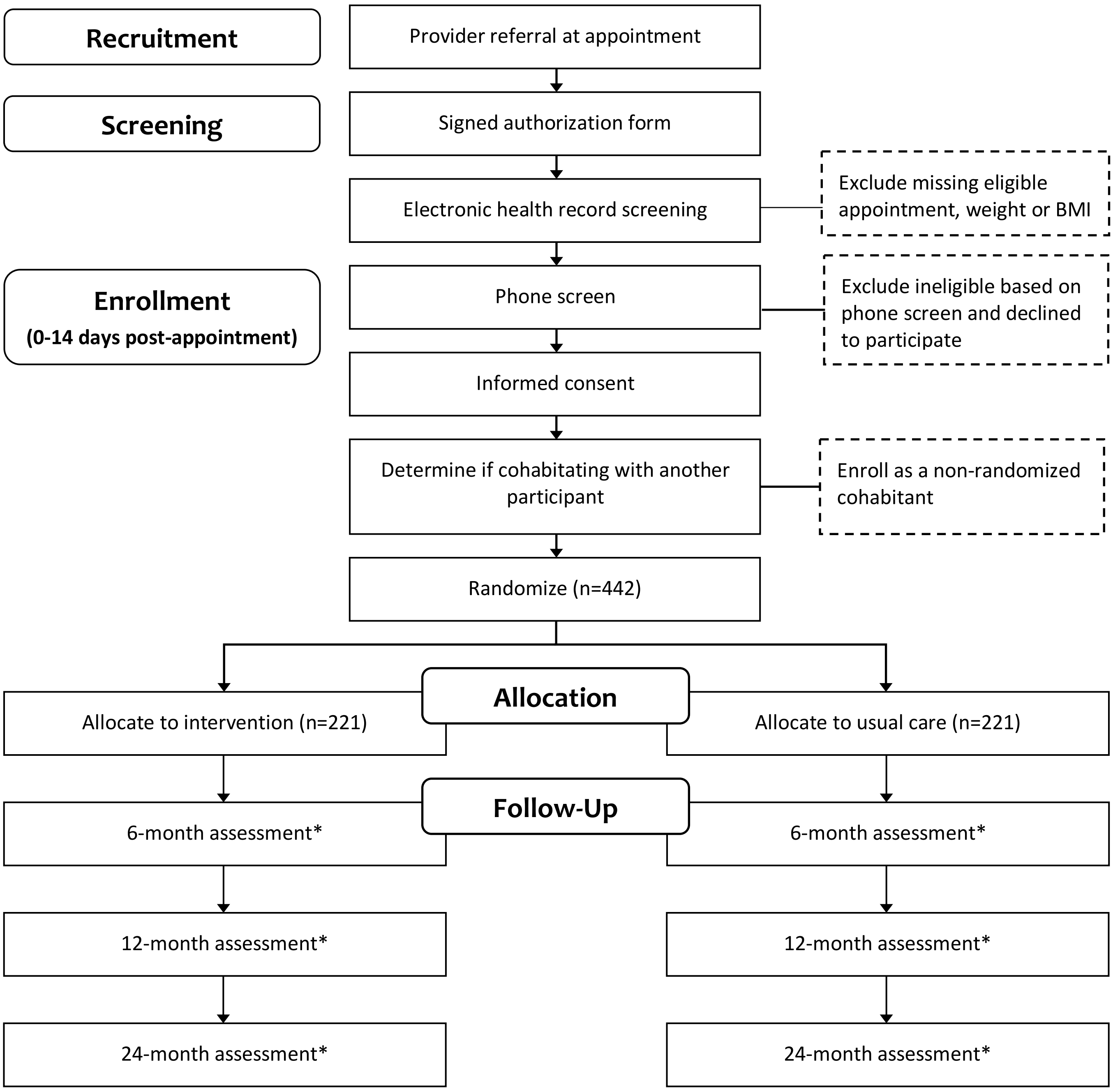

* Follow-up assessment data will be collected from the patients' electronic medical records after 24-months and analyzed with respect to the specified time points.
