# Supplementary material for: The Balance protocol: a pragmatic weight gain prevention randomized controlled trial for medically vulnerable patients within primary care
Source: BMC Public Health. 2019 May 17;19:596. doi: 10.1186/s12889-019-6926-7 (PMC6525404; doi:10.1186/s12889-019-6926-7)
Supplement: Supplementary file 2 — Script of a sample IVR call. This diagram depicts a script of a sample interactive voice response call used by participants to track weekly behavior changes goals via phone. (DOCX 45 kb) [file 12889_2019_6926_MOESM2_ESM.docx]

If 1, Play GOAL 1 Question File

**Voice File Played to Participant**

*(In English or Spanish)*

**INTRODUCTION**

“Hello, this is Julia. I'm calling from Project Balance. Balance is designed to help you make small changes to help you feel your best. These weekly calls help you and your Balance team keep track of your progress. This call should take about 3 minutes. Are you ready? Please press 1 for Yes and 2 for No.”

**GOAL 4 FEEDBACK & COMPOSITE RELATIVE FEEDBACK**

“You said [X] days. [GOAL 4 FEEDBACK]. Thank you for speaking with me today.

I will call you again next week. Goodbye.”

Play customized GOAL 1 feedback and then GOAL 2 question files.

Scoring for GOAL 1 is calculated by system

in real-time

Calculate average of all 4 scores, then compare average to last week’s average. Play appropriate GOAL 4 feedback and relative feedback files.

**Next Voice File & Scoring Logic**

Play customized GOAL 2 feedback and then GOAL 3 question files.

If 2, Play Call Back Later File: “Okay, I’ll call back later. Good-bye.”

Continue Feedback and

GOAL Questions until completes all GOAL 4 Questions and Feedback

Save data and end call.

Scoring for GOAL 2 is calculated by system

in real-time

**GOAL 1 QUESTION**

“First, we're going to ask you about [GOAL 1]. Use a number on your keypad to enter how many days this week you [GOAL 1]. Please enter a number between 0 and 7.”

**GOAL 1 FEEDBACK & GOAL 2 QUESTION**

“You said [X] days. [GOAL 1 FEEDBACK].

Next, we’re going to ask you about [GOAL 2] Use a number on your keypad to enter how many days you [GOAL 2] Please enter a number between 0 and 7.”
